# Polymorphisms of pri-miR-219-1 are associated with the susceptibility and prognosis of non-small cell lung cancer in a Northeast Chinese population

**DOI:** 10.18632/oncotarget.17035

**Published:** 2017-04-11

**Authors:** Chang Zheng, Xuelian Li, Lingzi Xia, Xue Fang, Xiaowei Quan, Zhihua Yin, Yuxia Zhao, Baosen Zhou

**Affiliations:** ^1^ Department of Epidemiology, School of Public Health, China Medical University, Shenyang, China; ^2^ Key Laboratory of Cancer Etiology and Prevention, China Medical University, Liaoning Provincial Department of Education, Liaoning, China; ^3^ Department of Radiotherapy, The Fourth Affiliated Hospital of China Medical University, Shenyang, China

**Keywords:** SNP, miRNA, NSCLC, susceptibility, prognosis

## Abstract

Occurrence and development of non-small cell lung cancer (NSCLC) is a complex process affected both by gene and environment. Single nucleotide polymorphisms (SNPs) in microRNAs’ (miRNAs) biogenesis influenced the expression of mature miRNAs, further had an impact on risk of NSCLC. Our study focused on the correlation between rs213210, rs421446 or rs107822 polymorphisms in pri-miR-219-1 and susceptibility or prognosis of NSCLC in Chinese. A case-control study of 405 new-diagnosis patients and 405 controls was performed. Ten ml venous blood from each subject was collected for genotype test via using TaqMan allelic discrimination methodology and SPSS was performed for statistical analyses. We found that CC genotype in rs213210 (OR=3.462, 95%CI=2.222-5.394, *P*<0.001) compared with TT genotype and GG genotype in rs107822 (OR=3.553, 95%CI=2.329-5.419, *P*<0.001) compared with AA genotype showed significantly increased risk of NSCLC. Haplotype analysis showed that pri-miR-219-1 haplotype C_rs213210_C_rs421446_G_rs107822_ was a dangerous haplotype for lung cancer. And polymorphisms in pri-miR-219-1 have showed no relationship with overall survival of NSCLC. Overall, these findings firstly showed that rs213210 and rs107822 could be meaningful as genetic markers for lung cancer risk.

## INTRODUCTION

Non-small cell lung cancer (NSCLC) accounts for approximately 80% of lung cancer [1], which ranks first for mortality among the malignant neoplasms [2], even with advanced chemotherapy and precise molecular-targeted treatment. According to histological type, NSCLC is divided into three main categories: squamous-cell carcinoma (SCC), adenocarcinoma (ADC) and large-cell carcinoma (LCC) [3]. Although smoking is one of the established risk factors for NSCLC, a particular genetic alteration or genotype combination, especially the interaction of environmental factors and genes is also involved in the occurrence and development of this malignant neoplasm [4–6].

MicroRNAs (miRNAs) are a large family of small endogenous non-coding RNA in the length of 21-25 nucleotides, which can modulate the expression of target messenger RNA (mRNA) by mRNA cleavage or post-transcriptional inhibition [7, 8]. Mature miRNAs are produced from the stem loop structure of precursor miRNAs (pre-miRNAs) derived from a long primary miRNA (pri-miRNA) [9]. In terms of all the gene sequence, miRNAs are more likely to produce a single gene mutation [10]. Emerging in the pri-miRNAs, the single nucleotide polymorphisms (SNPs) influences the maturation of respective miRNAs, which then affects its mature miRNAs expression and interaction as a consequence [11–13]. In the field of NSCLC, SNPs in the pri-miRNAs may prognosticate the development of cancer susceptibility and prognosis as genetic detection markers [14].

Hsa-miR-219a-1 (miR-219-1), located on chromosome 6 (6p21), may exert an influence on lung cancer [15, 16]. Several SNPs in the pri-miRNA sequence of miR-219-1 have been recognized and studied, of which rs213210 was associated with poor prognosis in colorectal cancer [17], rs107822 reduced the risk of esophageal cancer [18], and rs421446 was correlated to the risk of hepatocellular carcinoma progression [19]. Given the linkage disequilibrium of these three loci has been reported and verified, we propose a hypothesis in the present study: rs213210, rs421446 and rs107822 in miR-219-1, as a SNP or a haplotype, may be related to NSCLC susceptibility and prognosis.

## RESULTS

### Study characteristics

The demographic characteristics of 405 cases and 405 controls in the present study are summarized in Table 1. There was no significant difference in the distributions of age (*P*=0.49) between the cases (51.27±22.08 years) and controls (50.23±21.02 years). While, the distributions of gender and smoking status were of significant differences (both were *P*<0.001), so the next study was adjusted by smoking status, age and gender. Among 405 patients there were 324 subjects have follow-up information, with the median follow-up time was 24.35 months.

**Table 1:** Demographics of NSCLC patients and controls

		Cases	Controls	*P*-value
Gender	male	256(64.6)	140(35.4)	
	female	149(36.0)	265(64.0)	<0.001
Age(years)	mean±SD	51.27±22.08	50.23±21.02	0.49
Smoke	never	310(76.5)	357(88.1)	
	yes	95(23.4)	48(11.9)	<0.001
Histology	ADC	261(80.5)		
	SCC	69(21.3)		
	LCC	10(3.1)		
	unknown	65(20.1)		
Chemo	no	6(1.8)		
	yes	297(91.7)		
	unknown	21(6.5)		
Surgical	No	90(27.8)		
	yes	226(69.7)		
	unknown	8(2.5)		
Clinic stage	IA+IB	128(40.5)		
	IIA+IIB	77(24.8)		
	IIIC+IIID	58(17.9)		
	IV	61(18.8)		
Time(months)	mean±SD	24.35±18.70		
Status	alive	65(20.0)		
	dead	259(80.0)		

### pri-miR-219-1 polymorphisms are associated with the risk for NSCLC

Table 2 described genotype and allele distributions of rs213210 T>C, rs421446 C>T and rs107822 A>G in cases and controls. The observed genotype frequencies for three SNPs in the control were conformed to Hardy-Weinberg equilibrium (*P*>0.05). The minor allele frequencies (MAF) of rs213210, rs421446 and rs107822 were 0.42, 0.40 and 0.47, respectively. Both CC genotype carriers (adjusted OR=3.462, 95%CI=2.222-5.394, *P*<0.001) compared with TT genotype carriers in rs231210 and GG genotype carriers (adjusted OR=3.553, 95%CI=2.329-5.419, *P*<0.001) compared with AA genotype carriers in rs107822 demonstrated more increased risk of NSCLC. The results of stratified analysis by gender and smoking status showed in Supplementary Tables 1 and 2.

**Table 2:** Distribution of pri-miR-219-1 rs213210, rs421446 and rs107822 genotypes and ORs for NSCLC cases and controls

Genotype	Cases	Controls	*P*-value	OR	95%CI	*P* adj	OR adj	95%CI adj
rs213210								
TT	113	160	<0.001			<0.001		
CT	194	191	0.023	1.438	(1.052, 1.967)	0.057	1.383	(0.990, 1.932)
CC	98	54	<0.001	2.570	(1.705, 3.873)	<0.001	3.462	(2.222, 5.394)
TT	113	160						
CT+CC	292	245	0.001	1.688	(1.257, 2.266)	<0.001	1.779	(1.301, 2.432)
TT+CT	307	351						
CC	98	54	<0.001	2.075	(1.439, 2.991)	<0.001	2.874	(1.930, 4.279)
T allele	420	511						
C allele	390	299	<0.001	1.587	(1.301,1.935)	<0.001	1.792	(1.449, 2.216)
rs421446								
CC	129	174	0.004			0.040		
TC	198	170	0.004	1.571	(1.156, 2.134)	0.017	1.485	(1.075, 2.051)
TT	78	61	0.008	1.725	(1.150, 2.586)	0.079	1.469	(0.956, 2.258)
CC	129	174						
CT+TT	276	231	0.001	1.602	(1.202, 2.136)	0.522	0.920	(0.712, 1.188)
CC+CT	327	344						
TT	78	61	0.165	1.295	(0.899, 1.865)	0.012	1.475	(1.089, 1.997)
C allele	456	518						
T allele	354	292	0.002	1.377	(1.128,1.681)	0.026	1.272	(1.030, 1.572)
rs107822								
AA	79	162	<0.001			<0.001		
GA	206	174	<0.001	2.428	(1.735, 3.398)	<0.001	2.376	(1.664, 3.392)
GG	120	69	<0.001	3.566	(2.391, 5.320)	<0.001	3.553	(2.329, 5.419)
AA	79	162						
GA+GG	326	243	<0.001	2.751	(2.005, 3.774)	<0.001	2.711	(1.941, 3.788)
AA+GA	285	336						
GG	120	69	<0.001	2.050	(1.466, 2.868)	<0.001	2.074	(1.455, 2.957)
G allele	446	312						
A allele	364	498	<0.001	4.026	(3.251, 4.985)	<0.001	1.952	(1.582, 2.407)

*P*_adj_ is adjusted by smoking, age and gender.

### Interactions between polymorphisms of pri-miR-219-1 and tobacco exposure

This study further investigated the interaction of tobacco exposure and SNPs in cross-over analysis (Table 3). Relative to rs213210 non-smoking and TT genotype carriers, the OR (4.33) for CC genotype carriers with tobacco exposure was higher than the OR (2.45) for rs213210 TT carriers with tobacco exposure or the OR (3.83) for rs213210 CC genotype carriers without tobacco exposure. Similar results were obtained when rs421446- and rs107822-tobacco exposure were examined. Above cross-over results indicated that SNPs-tobacco exposure interaction may exist, therefore statistical tests were used to evaluate the significance of the interaction on both additive scale and multiplicative scale (data not shown). The results suggested that interactions between the SNPs and tobacco exposure were not significant on an additive scale or a multiplicative scale.

**Table 3:** Interaction of pri-miR-219-1 variants and tobacco exposure on NSCLC

Genotype	Smoking	Cases	Controls	*P*-value	OR(95% CI)	*P*-value adj.	OR(95% CI) adj.
rs213210							
TT	-	88	138	<0.001		<0.001	
CT	-	140	173	0.180	1.27(0.90, 1.80)	0.199	1.27(0.88, 1.84)
CC	-	82	46	<0.001	2.80(1.78, 4.38)	<0.001	3.83(2.37, 6.20)
TT	+	25	22	0.073	1.78(0.95, 3.35)	0.009	2.45(1.25, 4.81)
CT	+	54	18	<0.001	4.71(2.59, 8.54)	<0.001	5.15(2.74, 9.67)
CC	+	16	8	0.012	3.14(1.29, 7.64)	0.002	4.33(1.70, 11.06)
rs421446							
CC	-	109	150	<0.001		<0.001	
TC	-	148	154	0.101	1.32(0.95, 1.85)	0.179	1.27(0.90, 1.80)
TT	-	53	53	0.168	1.38(0.87, 2.17)	0.358	1.25(0.78, 2.01)
CC	+	20	24	0.676	1.15(0.60, 2.18)	0.453	1.30(0.66, 2.55)
TC	+	50	16	<0.001	4.30(2.33, 7.95)	<0.001	4.65(2.45, 8.82)
TT	+	25	8	0.001	4.30(1.87, 9.90)	0.001	4.30(1.81, 10.24)
rs107822							
AA	-	65	147	<0.001		<0.001	
GA	-	151	151	<0.001	2.26(1.56, 3.27)	<0.001	2.32(1.58, 3.42)
GG	-	94	59	<0.001	3.60(2.33, 5.58)	<0.001	3.64(2.30, 5.75)
AA	+	14	15	0.062	2.11(0.96, 4.63)	0.050	2.28(1.00, 5.22)
GA	+	55	23	<0.001	5.41(3.07, 9.54)	<0.001	6.06(3.33, 11.03)
GG	+	26	10	<0.001	5.88(2.68, 12.90)	<0.001	7.24(3.19, 16.44)

*P*-value adj. is adjusted by age and gender.

### Haplotype association analysis of SNPs on pri-miR-219-1

**Figure 1:**
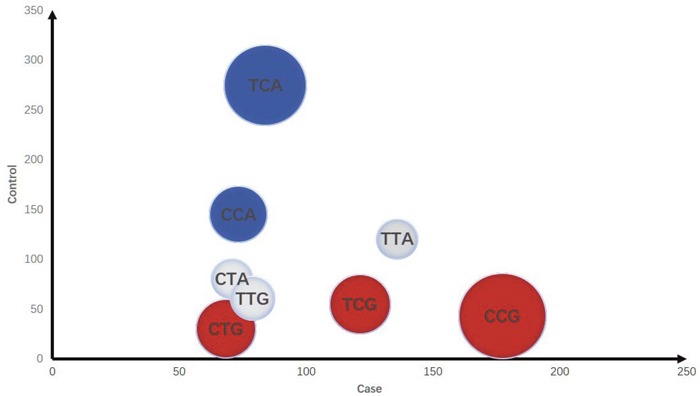
Distribution of haplotypes in cases and controls (1) The area of the pie represents the odd ratio (take the inverse when OR < 1). (2) Red pies present dangerous type, the blue ones are protectors, grey pie means no statistical significance different. (3) The axis represents the number of subjects.

The LD of rs213210, rs421446 and rs107822 was observed in Ensemble variation resources [20], each of them suggested great LD in pri-miR-219-1 of NSCLC (all D=1 and r^2^>0.5). The haplotype analysis of miR-219 SNPs was performed using online software SHEsis (http://analysis.bio-x.cn). Figure 1 intuitively represented the odd ratio of the dangerous and protective haplotype symbolized by the pie area. Each pie represented a haplotype, plotted by cases on horizontal axis, controls on vertical axis. Red pies meant dangerous haplotypes, blue pies meant protective haplotypes, and grey pies meant that distribution of these haplotypes was of no significant difference between cases and controls. The biggest red pie presented the risk of individual carrying C_rs213210_C_rs421446_G_rs107822_ was significantly higher than all other haplotypes (OR=4.997, 95%CI= 3.524-7.086, *P*<0.001) (Table 4).

**Table 4:** Association analysis of rs213210, rs421446 and rs107822 on the pri-miR-219-1 with risk of NSCLC

Haplotype	Cases (%)	Controls (%)	*P*-value	OR (95%CI)
C C A	73 (9.0)	145(17.9)	<0.001	0.456 (0.338, 0.615)
C C G	177(21.9)	43(5.3)	<0.001	4.997 (3.524, 7.086)
C T A	70(8.7)	80(9.9)	0.403	0.867 (0.620, 1.212)
C T G	68(8.5)	30(3.7)	<0.001	2.379 (1.533, 3.693)
T C A	83(10.4)	274(33.9)	<0.001	0.225 (0.172, 0.295)
T C G	121(15.0)	54(6.8)	<0.001	2.419 (1.730, 3.382)
T T A	135(16.8)	120(14.9)	0.291	1.155 (0.884, 1.509)
T T G	78(9.7)	60(7.5)	0.109	1.330 (0.937, 1.887)

### Evaluated the effects of pri-miR-219-1 SNPs in NSCLC clinical outcome

Table 5 rendered the results using multivariate Cox model for investigating the association between genotype polymorphisms in pri-miR-219 and NSCLC prognosis, adjusted for smoking status, age and gender. There was no relationship between rs213210, rs421446 or rs107822 polymorphisms and prognosis of OS in this study.

**Table 5:** pri-miR-219-1 SNPs associated with outcomes of NSCLC patients and stratified by stages

Genotype	Cases	*P*-value	HR	95%CI	*P*-value adj	HR adj	95%CI adj
rs213210							
TT	85	0.235			0.154		
CT	148	0.093	1.298	(0.957, 1.760)	0.063	1.344	(0.984, 1.836)
CC	91	0.224	1.236	(0.879, 1.737)	0.120	1.330	(0.928, 1.905)
Stage I+II							
T allele	206						
C allele	204	0.114	0.838	(0.673, 1.043)	0.184	0.860	(0.678, 1.075)
Stage III+IV							
T allele	112						
C allele	126	0.047	1.365	(1.004, 1.855)	0.065	1.358	(0.981, 1.878)
rs421446							
CC	110	0.358			0.413		
TC	151	0.152	0.818	(0.622, 1.077)	0.185	0.829	(0.627, 1.094)
TT	63	0.477	0.880	(0.617, 1.253)	0.482	0.878	(0.611, 1.261)
Stage I+II							
C allele	235						
T allele	175	0.310	0.892	(0.714, 1.113)	0.199	0.864	(0.692, 1.080)
Stage III+IV							
C allele	136						
T allele	102	0.102	0.775	(0.572, 1.051)	0.147	0.795	(0.582, 1.084)
rs107822							
AA	66	0.147			0.139		
GA	159	0.693	1.066	(0.776, 1.465)	0.536	1.107	(0.803, 1.526)
GG	99	0.084	1.355	(0.960, 1.913)	0.065	1.384	(0.980, 1.955)
Stage I+II							
A allele	192						
G allele	217	0.054	1.243	(0.996, 1.552)	0.057	1.241	(0.994, 1.549)
Stage III+IV							
A allele	99						
G allele	140	0.895	0.980	(0.724, 1.326)	0.930	0.987	(0.729, 1.336)

*P*_adj_ is adjusted by smoking, age and gender.

## DISCUSSION

The occurrence and development of NSCLC is a complicated process, which is inevitably affected by multiple established or uncertain factors. In recent years, researchers all around world have made great efforts to elucidate microRNAs’ functions in initiation and progression of lung cancer. Recent studies showed that expression level of miRNAs in cells from cancer tissue were often in deregulation compared with adjacent normal tissues [13, 21]. An early group has suggested that miR-219 was significantly downregulated in hepatocellular carcinoma, also exerted tumor-suppressive effects in hepatic carcinogenesis via inhibiting transcription and translation of glypican-3 [22].

SNPs in microRNAs non-coding region may affect Drosha or Dicer trimming or reshaping miRNA target genes. A previous study on esophageal squamous cell carcinoma in Chinese Kazakh demonstrated that rs107822 A allele in primary form of miR-219-1 may reduce the efficacy of the maturation process from pre-mir-219-1 to miR-219-1 compared with G allele [18].

On the whole, the present research firstly reported association between the polymorphisms in miR-219-1 (rs213210, rs421446 and rs107822) and susceptibility or prognosis of NSCLC. In our study, after Bonferroni correction, CC genotype carriers of rs213210, as well as GG and GA genotypes carriers of rs107822 were still significantly dangerous for risk of lung cancer, compared with other genotypes. A previous study reported that rs107822 was associated with risk of esophageal squamous cell carcinoma in Chinese Kazakh [18], moreover, this locus also influenced susceptibility of schizophrenia via N-Methyl-D-aspartate-type glutamate receptor signaling pathway [23, 24]. Taken together, these results suggested that rs107822 polymorphism may play a considerable role in the process of cancer development.

Given that smoking is an established major environmental risk for developing lung cancer, gene-environment interactions may have potential effect. An early study to evaluate the lungs of environmental cigarette smoke-exposed mice showed that expression level of miR-219-1-5p was significantly low compared with normal mice [25]. The effects of interaction between polymorphisms in miR-219-1 and tobacco exposure on lung cancer have seldom been investigated. Hence, in our study, we evaluated the interaction between smoking status and three miRNA SNPs by using cross-over analysis. We found that tobacco exposure and SNPs interaction may exist, however, not significant in an additive scale or a multiplicative scale.

Haplotypes are more meaningful than a single SNP for changes in gene function [26, 27]. We found that C_rs213210_ C_rs421446_G_rs107822_ haplotype among 5 common haplotypes in our study was most dangerous than any other haplotypes. However, SNPs and haplotypes of miR-219-1 with susceptibility to lung cancer should be investigated in further studies with a larger size of samples.

In Cox model, there were no significant associations between the polymorphism in miR-219-1 (rs213210, rs421446 and rs107822) and survival of NSCLC patients. Results of rs213210 were consistent with those from a previous study [14]. But in stage III of colorectal cancer, C allele carriers in rs213210 were significantly related with a better outcome [28].

Although we still cannot fully understand the mechanism of NSCLC, it is needless to say the importance of research that SNPs affect susceptibility and prognosis of NSCLC. Considering the temporality of epigenetic change and diverse distribution of miR-219-1 in human body, there could be differences in the distribution of SNPs loci between tissues and blood. Although statistical power is 87% in our study, a larger sample size is required to verify the relation of rs213210, rs421446 or rs107822 polymorphisms with the mechanism of lung cancer in the further study.

## MATERIALS AND METHODS

### Subject data collection

Our hospital-based case control study population consisted of 405 NSCLC patients and 405 cancer-free controls. Cases were firstly diagnosed as NSCLC by the professional pathologists without restriction of gender or histology. Meanwhile, control subjects as frequency-matched cases according to age (±5 years) were individuals with other type of diseases, such as gastritis, coronary disease, diabetes mellitus and so on. The whole subjects were recruited from unrelated ethnic Han Chinese, with at least two years of follow-up time, in the Fourth Affiliated Hospital of China Medical University. Our study was performed in full compliance with requirements of the Institutional Review Board of China Medical University. Each subject signed an informed consent form. And consent from subject's representative was approved if subject's consent could not be obtained.

The data was collected under the following strict criterions: (i) 10 ml peripheral blood was donated by each participant during their first hospitalization; (ii) Each volunteer have no history of radiotherapy or chemotherapy; (iii) Tobacco exposure (a non-smoker is defined less than 100 cigarettes consumed in his whole life, otherwise are smokers) and demographical characteristics were questioned by well-trained interviewers under a pretested questionnaire just after taking their blood; and (iv) Patient's pathology and medical records were reviewed by us for clinical stage, pathologic type, date of diagnosis and performance status, twice a year.

### DNA genotyping

Genomic DNA samples were extracted from proteinase K-digested leukocyte pellet by conventional phenol–chloroform extraction and ethanol precipitation method [29]. SNP Genotyping was done using Taqman allelic discrimination assay from Applied Biosystems (ABI, Foster City, CA). The SNP Assays were also purchased from ABI, including primer and FAM/VIC probe (assay ID C_2215074_10 for rs213210, C_27015692_10 for rs421446 and C_2215075_20 for rs107822). Each reaction in Fast 96-well plate was conducted in a total volume of 10 μL including 2 μL purified genomic DNA (15–25 ng/μL), 5 μL TaqMan Genotyping master mix, 2.5 μL RNase-free water and 0.5 μL SNP Assay. The quantitative real-time PCR (qPCR) condition was as follows: 95°C for 10 min, followed by 47 cycles of 30 secs at 92°C, and 1 min at 62°C. The reacted results were read by an ABI 7500 FAST Real-Time PCR System with the Sequence Detection Software. A 5% selected sample were duplicated to validate the results, which were 100% consistent.

### Statistical analysis

The χ2 test and Student's *t*-test were used to examine difference between cases and controls in demographic variables, tobacco exposure and SNPs of genotypes. Hardy–Weinberg equilibrium (HWE) [30] of each SNP was tested by Pearson's goodness-of-fit test among control. Logistic regression model and Cox's proportional hazards model were used to estimate the odds ratios (ORs) and hazard ratios (HRs) and their 95% confidence intervals (CIs) for unconditional logistic regression analysis and the multivariate survival analyses, respectively. Crossover analysis was applied to examine gene-environment interactions. All the analyses were adjusted by age and gender. Linkage disequilibrium (LD) was measured in Haploview algorithm [31], haplotype analysis were performed by using SHEsis Software [32]. All above analyses were two-sided, and performed in Statistical Products and Services Solutions software (v.16.0, SPSS Institute Cary, Chicago, IL, USA) unless specified. A value of *P*< 0.05 was considered statistical significant.

## CONCLUSION

There may exist significant association between polymorphisms in miR-219-1 (rs213210 and rs107822) and lung cancer risk in Chinses population. There is no relationship between polymorphisms in miR-219-1 (rs213210, rs421446 and rs107822) and outcome of lung cancer.

## SUPPLEMENTARY MATERIALS TABLES



## References

[1] Govindan R, Page N, Morgensztern D, Read W, Tierney R, Vlahiotis A, Spitznagel EL, Piccirillo J (2006). Changing epidemiology of small-cell lung cancer in the United States over the last 30 years: analysis of the surveillance, epidemiologic, and end results database. J Clin Oncol.

[2] Wang H, Wu S, Zhao L, Zhao J, Liu J, Wang Z (2015). Clinical use of microRNAs as potential non-invasive biomarkers for detecting non-small cell lung cancer: a meta-analysis. Respirology.

[3] Wistuba II (2007). Genetics of preneoplasia: lessons from lung cancer. Curr Mol Med.

[4] Ren YW, Yin ZH, Wan Y, Guan P, Wu W, Li XL, Zhou BS (2013). P53 Arg72Pro and MDM2 SNP309 polymorphisms cooperate to increase lung adenocarcinoma risk in Chinese female non-smokers: a case control study. Asian Pac J Cancer Prev.

[5] Albright F, Teerlink C, Werner TL, Cannon-Albright LA (2012). Significant evidence for a heritable contribution to cancer predisposition: a review of cancer familiality by site. BMC Cancer.

[6] Brennan P, Hainaut P, Boffetta P (2011). Genetics of lung-cancer susceptibility. Lancet Oncol.

[7] Lee Y, Jeon K, Lee JT, Kim S, Kim VN (2002). MicroRNA maturation: stepwise processing and subcellular localization. EMBO J.

[8] Bartel DP (2004). MicroRNAs: genomics, biogenesis, mechanism, and function. Cell.

[9] Zeng Y, Yi R, Cullen BR (2005). Recognition and cleavage of primary microRNA precursors by the nuclear processing enzyme Drosha. EMBO J.

[10] Sun G, Yan J, Noltner K, Feng J, Li H, Sarkis DA, Sommer SS, Rossi JJ (2009). SNPs in human miRNA genes affect biogenesis and function. RNA.

[11] Slaby O, Bienertova-Vasku J, Svoboda M, Vyzula R (2012). Genetic polymorphisms and microRNAs: new direction in molecular epidemiology of solid cancer. J Cell Mol Med.

[12] Papurica M, Rogobete AF, Sandesc D, Cradigati CA, Sarandan M, Crisan DC, Horhat FG, Boruga O, Dumache R, Nilima KR, Nitu R, Stanca H, Bedreag OH (2016). The expression of nuclear transcription factor kappa B (NF-kappaB) in the case of critically ill polytrauma patients with sepsis and its interactions with microRNAs. Biochem Genet.

[13] Nitu R, Rogobete AF, Gundogdu F, Tanasescu S, Boruga O, Sas A, Popovici SE, Hutanu D, Pilut C, Sarau CA, Candea AC, Stan AT, Moise LM (2017). microRNAs expression as novel genetic biomarker for early prediction and continuous monitoring in pulmonary cancer. Biochem Genet.

[14] Yoon KA, Yoon H, Park S, Jang HJ, Zo JI, Lee HS, Lee JS (2012). The prognostic impact of microRNA sequence polymorphisms on the recurrence of patients with completely resected non-small cell lung cancer. J Thorac Cardiovasc Surg.

[15] Wang Y, Broderick P, Webb E, Wu X, Vijayakrishnan J, Matakidou A, Qureshi M, Dong Q, Gu X, Chen WV, Spitz MR, Eisen T, Amos CI (2008). Common 5p15.33 and 6p21.33 variants influence lung cancer risk. Nat Genet.

[16] de Mello RA, Ferreira M, Soares-Pires F, Costa S, Cunha J, Oliveira P, Hespanhol V, Reis RM (2013). The impact of polymorphic variations in the 5p15, 6p12, 6p21 and 15q25 loci on the risk and prognosis of portuguese patients with non-small cell lung cancer. PLoS One.

[17] Pardini B, Rosa F, Naccarati A, Ye Y, Wu X, di Gaetano C, Buchler T, Novotny J, Matullo G, Vodicka P (2015). Polymorphisms in microRNA genes as predictors of clinical outcomes in colorectal cancer patients. Carcinogenesis.

[18] Song X, You W, Zhu J, Cui X, Hu J, Chen Y, Liu W, Wang L, Li S, Wei Y, Yang L, Li F (2015). A genetic variant in miRNA-219-1 is associated with risk of esophageal squamous cell carcinoma in Chinese Kazakhs. Dis Markers.

[19] Cheong JY, Shin HD, Kim YJ, Cho SW (2013). Association of polymorphism in MicroRNA 219-1 with clearance of hepatitis B virus infection. J Med Virol.

[20] Chen Y, Cunningham F, Rios D, McLaren WM, Smith J, Pritchard B, Spudich GM, Brent S, Kulesha E, Marin-Garcia P, Smedley D, Birney E, Flicek P (2010). Ensembl variation resources. BMC Genomics.

[21] Li S, Gao M, Li Z, Song L, Gao X, Han J, Wang F, Chen Y, Li W, Yang J, Han X (2016). Role of microRNAs in metastasis of non-small cell lung cancer. Front Biosci (Landmark Ed).

[22] Huang N, Lin J, Ruan J, Su N, Qing R, Liu F, He B, Lv C, Zheng D, Luo R (2012). MiR-219-5p inhibits hepatocellular carcinoma cell proliferation by targeting glypican-3. FEBS Lett.

[23] Zhang Y, Fan M, Wang Q, He G, Fu Y, Li H, Yu S (2015). Polymorphisms in microRNA genes and genes involving in NMDAR signaling and schizophrenia: a case-control study in Chinese Han population. Sci Rep.

[24] Sun YJ, Yu Y, Zhu GC, Sun ZH, Xu J, Cao JH, Ge JX (2015). Association between single nucleotide polymorphisms in MiR219-1 and MiR137 and susceptibility to schizophrenia in a Chinese population. FEBS Open Bio.

[25] Izzotti A, Calin GA, Arrigo P, Steele VE, Croce CM, De Flora S (2009). Downregulation of microRNA expression in the lungs of rats exposed to cigarette smoke. FASEB J.

[26] Yin Z, Cui Z, Guan P, Li X, Wu W, Ren Y, He Q, Zhou B (2015). Interaction between polymorphisms in pre-MiRNA genes and cooking oil fume exposure on the risk of lung cancer in Chinese non-smoking female population. PLoS One.

[27] Yin Z, Cui Z, Ren Y, Zhang H, Yan Y, Zhao Y, Ma R, Wang Q, He Q, Zhou B (2014). Genetic polymorphisms of TERT and CLPTM1L, cooking oil fume exposure, and risk of lung cancer: a case-control study in a Chinese non-smoking female population. Med Oncol.

[28] Lin M, Gu J, Eng C, Ellis LM, Hildebrandt MA, Lin J, Huang M, Calin GA, Wang D, Dubois RN, Hawk ET, Wu X (2012). Genetic polymorphisms in microRNA-related genes as predictors of clinical outcomes in colorectal adenocarcinoma patients. Clin Cancer Res.

[29] Kerkhof L, Ward BB (1993). Comparison of nucleic acid hybridization and fluorometry for measurement of the relationship between RNA/DNA ratio and growth rate in a marine bacterium. Appl Environ Microbiol.

[30] Wigginton JE, Cutler DJ, Abecasis GR (2005). A note on exact tests of Hardy-Weinberg equilibrium. Am J Hum Genet.

[31] Barrett JC, Fry B, Maller J, Daly MJ (2005). Haploview: analysis and visualization of LD and haplotype maps. Bioinformatics.

[32] Shi YY, He L (2005). SHEsis, a powerful software platform for analyses of linkage disequilibrium, haplotype construction, and genetic association at polymorphism loci. Cell Res.

