# Supplementary material for: Polymorphisms of pri-miR-219-1 are associated with the susceptibility and prognosis of non-small cell lung cancer in a Northeast Chinese population
Source: Oncotarget. 2017 Apr 11;8(34):56533–41. doi: 10.18632/oncotarget.17035 (PMC5593580; doi:10.18632/oncotarget.17035)
Supplement: Supplementary file 1 [file oncotarget-08-56533-s001.pdf]

Polymorphisms of pri-miR-219-1 are associated with the susceptibility and prognosis of non-small cell lung cancer in a Northeast Chinese population

SUPPLEMENTARY TABLES

Supplementary Table 1: pri-miR-219-1 SNPs associated with risk of NSCLC and stratified by gender

Genotype	Cases	Controls	*P* value	OR	95%CI
rs213210					
Male					
TT	76	56	0.012		
CT	129	67	0.186	1.353	(0.864, 2.116)
CC	51	17	0.003	3.081	(1.466, 6.477)
Female					
TT	37	104	<0.001		
CT	65	124	0.151	1.429	(0.878, 2.326)
CC	47	37	<0.001	3.399	(1.959, 5.899)
rs421446					
Male					
CC	76	61	0.035		
TC	133	60	0.012	1.812	(1.140, 2.880)
TT	47	19	0.100	1.636	(0.910, 2.941)
Female					
CC	53	113	0.305		
TC	65	110	0.259	1.289	(0.830, 2.002)
TT	31	42	0.159	1.538	(0.845, 2.800)
rs107822					
Male					
AA	55	55	0.001		
GA	131	57	0.001	2.298	(1.413, 3.737)
GG	70	28	0.002	2.500	(1.405, 4.447)
Female					
AA	24	107	<0.001		
GA	75	117	<0.001	2.858	(1.684, 4.851)
GG	50	41	<0.001	5.437	(2.968, 9.960)

Supplementary Table 2: pri-miR-219-1 SNPs associated with risk of NSCLC and stratified by smoking status

Genotype	Cases	Controls	*P* value	OR	95%CI
rs213210					
Non-smokers					
TT	88	138	<0.001		
CT	140	173	0.180	1.269	(0.896, 1.797)
CC	82	46	>0.001	2.795	(1.784, 4.381)
Smokers					
TT	25	22	0.052		
CT	54	18	0.015	2.640	(1.207, 5.775)
CC	16	8	0.279	1.760	(0.632, 4.900)
rs421446					
Non-smokers					
CC	109	150	0.191		
TC	148	154	0.101	1.323	(0.947, 1.848)
TT	53	53	0.168	1.376	(0.874, 2.166)
Smokers					
CC	20	24	0.003		
TC	50	16	0.002	3.750	(1.655, 8.497)
TT	25	8	0.009	3.750	(1.389, 10.122)
rs107822					
Non-smokers					
AA	65	147	<0.001		
GA	151	151	<0.001	2.262	(1.564, 3.271)
GG	94	59	<0.001	3.603	(2.327, 5.579)
Smokers					
AA	14	15	0.074		
GA	55	23	0.035	2.562	(1.067, 6.152)
GG	26	10	0.051	2.786	(0.994, 7.809)
